# A Full-Spectrum Registration Method for Zhuhai-1 Satellite Hyperspectral Imagery

**DOI:** 10.3390/s20216298

**Published:** 2020-11-05

**Authors:** Jinjun Meng, Jiaqi Wu, Linlin Lu, Qingting Li, Qiang Zhang, Suyun Feng, Jun Yan

**Affiliations:** 1 Zhuhai Orbita Aerospace Science & Technology Co., Ltd., Zhuhai 519080, China; mengjj@myorbita.net (J.M.); jiaqiwu@myorbita.net (J.W.); zhangq@myorbita.net (Q.Z.); masy@myorbita.net (S.F.); yan@myorbita.net (J.Y.); 2 Key Laboratory of Digital Earth Science, Aerospace Information Research Institute, Chinese Academy of Sciences, Beijing 100094, China; 3 Airborne Remote Sensing Center, Aerospace Information Research Institute, Chinese Academy of Sciences, Beijing 100094, China; liqt@radi.ac.cn

**Keywords:** Zhuhai-1 satellite, hyperspectral image, scale-invariant feature transform, full-spectrums registration, Delaunay triangulation

## Abstract

Accurate registration is an essential prerequisite for analysis and applications involving remote sensing imagery. It is usually difficult to extract enough matching points for inter-band registration in hyperspectral imagery due to the different spectral responses for land features in different image bands. This is especially true for non-adjacent bands. The inconsistency in geometric distortion caused by topographic relief also makes it inappropriate to use a single affine transformation relationship for the geometric transformation of the entire image. Currently, accurate registration between spectral bands of Zhuhai-1 satellite hyperspectral imagery remains challenging. In this paper, a full-spectrum registration method was proposed to address this problem. The method combines the transfer strategy based on the affine transformation relationship between adjacent spectrums with the differential correction from dense Delaunay triangulation. Firstly, the scale-invariant feature transform (SIFT) extraction method was used to extract and match feature points of adjacent bands. The RANdom SAmple Consensus (RANSAC) algorithm and the least square method is then used to eliminate mismatching point pairs to obtain fine matching point pairs. Secondly, a dense Delaunay triangulation was constructed based on fine matching point pairs. The affine transformation relation for non-adjacent bands was established for each triangle using the affine transformation relation transfer strategy. Finally, the affine transformation relation was used to perform differential correction for each triangle. Three Zhuhai-1 satellite hyperspectral images covering different terrains were used as experiment data. The evaluation results showed that the adjacent band registration accuracy ranged from 0.2 to 0.6 pixels. The structural similarity measure and cosine similarity measure between non-adjacent bands were both greater than 0.80. Moreover, the full-spectrum registration accuracy was less than 1 pixel. These registration results can meet the needs of Zhuhai-1 hyperspectral imagery applications in various fields.

## 1. Introduction

The Zhuhai-1 hyperspectral satellite is a commercial remote sensing micro-nano satellite constellation that was launched and is currently operated by Zhuhai Orbita Aerospace Science and Technology Co., Ltd. It is the first satellite constellation constructed and operated by a private company in China. The Zhuhai-1 satellite constellation is planned to feature 34 small satellites, including video, hyperspectral, radar, high-resolution optical, and infrared satellites in different orbits. A total of twelve satellites have been launched so far and include four video satellites (Orbita Video Satellites (OVS)) and eight hyperspectral satellites (Orbita Hyperspectral Satellites (OHS)). These eight hyperspectral satellites can observe the Earth’s surface every 2.5 days and have become the world’s leading hyperspectral satellite constellation. The satellites’ hyperspectral imagery has been widely used in land and resource mapping, water quality monitoring, precision agriculture services, and other fields [1].

Image registration is an indispensable prerequisite for image analysis processes such as geometric correction, image fusion, and image stitching [2,3,4]. It is also an important initial step for remote sensing applications in fields such as change detection, environmental monitoring, crop yield estimation, mineral exploration, and so on [5,6,7,8,9]. The various image registration methods can be divided into two categories, including area-based registration and feature-based registration [10,11]. The area-based registration methods are mostly implemented by analyzing the grayscale distribution and histogram of images [12,13]. Their limitation lies in the difficulty in processing images with different grayscale characteristics. The feature-based registration method estimates the mapping relationship by extracting features such as feature points, lines, or edges, and the morphological regions of two images. An advantage of feature-based methods is their capability for handling the complex misalignments between scenes [14,15]. The scale-invariance feature transform (SIFT) method allows for the extraction of distinctive invariant features from images. This extraction method is robust against noise, geometric distortion, and radiometric differences, and has been extensively adopted for registration of optical imagery [16,17,18]. The SIFT descriptors were compared with steerable filters, differential invariants, complex filters, and moment invariants and performed best [19]. However, SIFT tends to obtain an insufficient number of matching point pairs and a low registration accuracy for multispectral images with intrinsic differences or images with illumination changes. For feature-based registration methods, robust fitting of global transformation models is very important. The RANdom SAmple Consensus (RANSAC) algorithm iteratively derives the parameters of a mathematic model from observation data that contains outliers [20,21]. Based on RANSAC, improved methods such as maximum likelihood estimation sample consensus and progressive sample consensus were further developed [22,23]. Due to their robustness and low computational complexity, the RANSAC and related methods were widely applied to outlier removal and fitting of transformation models for remote sensing image registration [24,25].

In recent years, graph theory has been introduced to establish a geometrical relationship between feature points in image registration [26]. Aguilar et al. first proposed a feature-graph-based point-matching algorithm that used local structural information [27]. Delaunay triangulation (DT) is an optimal planar partition that has been used to represent geometrical structures [28]. Zhao et al. adopted the graph structures from Delaunay triangulation based on the spatial triangulations among SIFT feature points. Moreover, they developed a novel dual-graph-based matching method for multispectral/multi-date image registration that significantly improved performance [29]. Zheng et al. proposed an accurate hyperspectral and infrared satellite image registration method that used the correspondences between local points (e.g., the Delaunay triangular edges and triangular surfaces) [30]. The constructed Delaunay triangular mesh enabled the estimation of transformation model parameters for each triangular facet, and was well adapted to the local distortion of satellite images.

The flutter and focal plane distortion in Zhuhai-1 OHS imagery is caused by an unstable positioning accuracy which is a result of the limitations in complementary metal-oxide semiconductor (CMOS) technology. As a result, inconsistent geometric positioning between different spectral bands of hyperspectral images is created during image processing. Jiang et al. conducted on-orbit inspections of the external and internal parameter errors produced by the Zhuhai-1 hyperspectral camera, where Digital orthophoto map and digital elevation model data was used as control data. They established the internal orientation element model (IOEM) for on-orbit geometric calibration [31]. However, the registration accuracy produced by the IOEM method is easily affected by the satellite attitude stability. Additionally, the gray levels can vary for the same land features in different spectral bands, since the hyperspectral images are obtained by spectral imagers with gradient filters. This makes it problematic to extract and match the feature points of non-adjacent spectral bands in OHS hyperspectral images. Therefore, accurate inter-band registration of Zhuhai-1 satellite hyperspectral images remains challenging. In this paper, a novel full-spectrum registration algorithm was proposed based on feature point matching methods. The algorithm combines the transference strategy based on the affine transformation relationship between adjacent bands with the differential correction from dense Delaunay triangulation. The performance of the proposed registration method was evaluated using the coordinate deviation of matching points, as well as the similarity measures for hyperspectral images with different topographies and roll angles.

## 2. Materials

**Figure 1:**
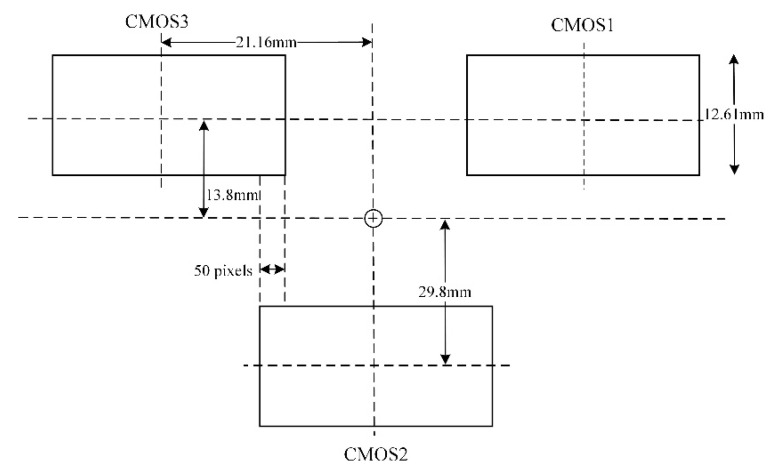
Focal plane arrangement for the hyperspectral camera.

**Figure 2:**
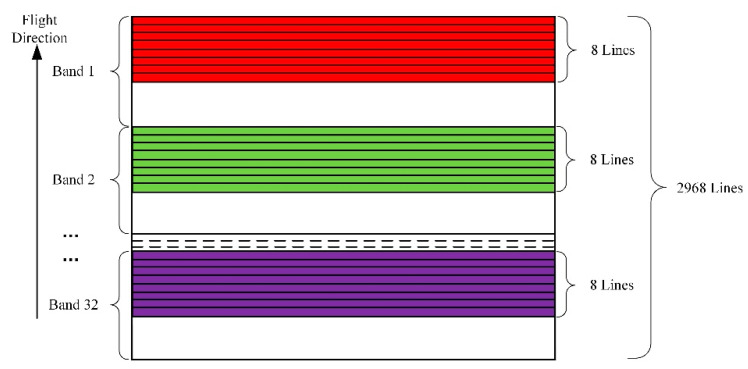
CMOS sensor spectrum distribution.

**Figure 3:**
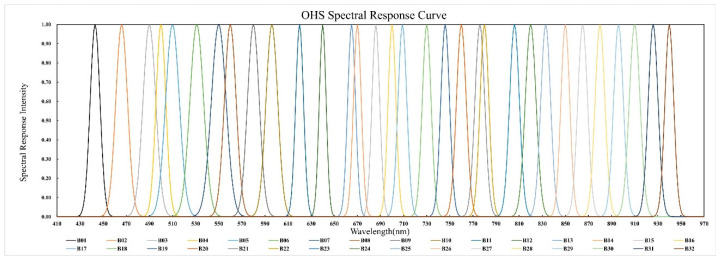
Spectral response functions of OHS hyperspectral bands.

In this study, the experimental imagery was acquired by the Zhuhai-1 hyperspectral satellite constellation (OHS-A, B, C, D) launched on 26 April 2018. The camera onboard the Zhuhai-1 hyperspectral satellite is spliced by three CMOS image sensors. The splicing method for the focal surface is illustrated in Figure 1. Figure 2 displays the spectral composition of each CMOS image sensor. Each CMOS image sensor is composed of 5056 × 2968 pixels, the size of each pixel is 4.25 μm, and the spectral range is 400–1000 nm. Each CMOS image sensor divides the 400–1000 nm spectrum into 32 spectral bands on average with a gradient filter. Each spectral band occupies about 92 lines on the sensor (2968 divided by 32). Only eight rows of pixels are used in each spectral band for eight-level integral imaging due to the low resolution and signal-to-noise ratio (Figure 2). The main parameters for the OHS and payload are displayed in Table 1. Figure 3 shows the spectral response functions of each band of OHS hyperspectral sensors. 

Three hyperspectral datasets with different topographies and roll angles were selected to evaluate the registration accuracy. The first dataset covers the southwestern part of Arizona, USA. The area consists of a large mountain basin with plain areas. The second dataset covers the central part of Chongzuo City, Guangxi Zhuang Autonomous Region, China. This area is crossed by the Zuojiang River and its tributaries and features both alluvial plains and hilly areas. The third dataset covers the northern part of Turpan City, Xinjiang Uygur Autonomous Region, China. The image shows the majestic Bogda Mountains, as well as the oasis plain belt that lies below sea level. The size of each image was 5056 × 5056 pixels. The experiment dataset parameters are listed in Table 2. 

## 3. Methods

**Figure 4:**
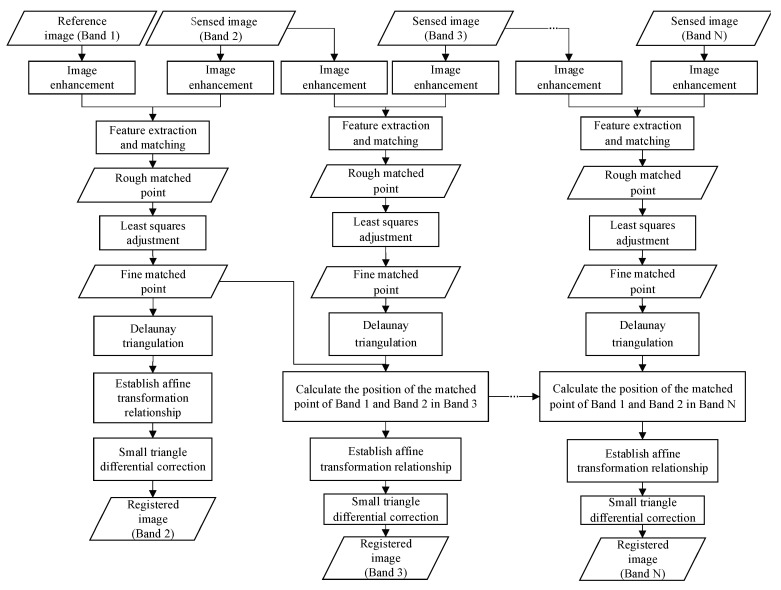
Full-spectrum registration workflow.

The proposed full-spectrum inter-band registration method consists of two parts, including relative and absolute registration. Relative registration involves matching feature points between adjacent spectral bands to obtain accurate matching points. Absolute registration involves selecting a spectral band as the reference image and performing registration for all other bands. This process involves transferring the affine transformation relationship between adjacent bands and differential correction from dense Delaunay triangulation. By comparing image quality evaluation metrics such as histogram, clarity, Shannon entropy, and Signal-to-Noise Ratio (SNR) of different bands, an image band with higher quality was selected as the reference image. Figure 4 displays the full-spectrum registration workflow used in this study.

### 3.1. Relative Registration

#### 3.1.1. Feature Matching of Adjacent Bands

The scale-invariant feature transform (SIFT) feature operator is a local image feature description operator that maintains invariance to image scaling, rotation, and affine transformation [32]. In this study, the feature points were extracted and matched based on SIFT features. The flow of the algorithm is described below.

1.  Keypoint detection

The image scale-space L(*x*, *y*, *σ*) was produced from the convolution of a variable-scale Gaussian function (G(*x*, *y*, *σ*)) with an input image (I(*x*, *y*)), as shown in the following equation:

L(*x*, *y*, *σ*) = G(*x*, *y*, *σ*)⊗I(*x*, *y*), (1)

where ⊗ is the convolution operation in *x* and *y*; G(*x*, *y*, *σ*) is a Gaussian function with a variable scale and (*x*, *y*) are pixel coordinates; and σ is the scale space factor, and its size determines the smoothness scale of the image. A large scale corresponds to general features in the image, while a small scale corresponds to detailed features.

Different scale space factors were used to construct a difference of Gaussian (DoG) pyramid based on the original image. A point was selected as the key point if it is the largest or smallest point within 26 neighborhoods of the same layer and the upper and lower layers in DoG scale space.

2.  Keypoint direction matching

An orientation histogram was formed from the gradient orientations of sample points within a region around the keypoint. The orientation histogram ranged from 0° to 360°, and each 10° represented a direction. A total of 36 directions were derived in this study. The peaks in the orientation histogram correspond to the dominant directions of local gradients at the keypoint.

3.  Keypoint descriptor

The local area around the feature point was rotated clockwise by θ (e.g., adjusted to 0°) when constructing the feature descriptor to ensure its rotation invariance. In the rotated region, the surrounding pixel region was partitioned with the keypoint as the center. The gradient accumulation value is calculated for eight directions on each sub-block, and a gradient direction histogram was then drawn. Finally, the feature vector representing local keypoint information could be obtained. Through the above steps, the influence of geometric factors such as scale change and rotation on the SIFT feature vector could be removed. Moreover, the influence of illumination change could be reduced if the SIFT feature vector is further normalized [33].

4.  Feature matching

The similarity of keypoints was measured based on Euclidean distance after the feature vectors are generated for the two images. The keypoint *p* can be taken from the reference image and used to search for the two keypoints *n* and *m* that have the closest Euclidean distance from *p* in the matched image. The Euclidean distance can then be calculated between *p* and *n* and *p* and *m*, respectively. The matching process is successful if the ratio of the closest distance to the next closest distance is less than a certain threshold.

5.  Elimination of mismatched points

The RANdom SAmple Consensus (RANSAC) algorithm was used to find the optimal mathematical model, where the number of matching point pairs is the maximum value. Hence, mismatched point pairs were eliminated. The basic steps of the algorithm are described as follows:

Step 1: Randomly select the minimum number of points required to determine the model parameters.

Step 2: Calculate the model parameters.

Step 3: Find how many points from the entire point set fit the model within a user defined tolerance of λ.

Step 4: Re-estimate the model parameters using identified inliers and terminate the process if the fraction of inlier points over the entire point set exceeds a predefined threshold ψ.

Step 5: Otherwise, repeat steps 1 through 4 for a prescribed number of iterations.

#### 3.1.2. Matching Point Adjustment

1.  Homography matrix estimation

The matching point pairs of two images are defined as $p_{1}\left(x_{1},y_{1}\right)$ and $p_{2}\left(x_{2},y_{2}\right)$, and their homography matrix H is given as:

(2) $$\left[\begin{array}{c} x_{2}\\ y_{2}\\ 1 \end{array}\right]=\left[\begin{array}{ccc} H_{11} & H_{12} & H_{13}\\ H_{21} & H_{22} & H_{23}\\ H_{31} & H_{32} & H_{33} \end{array}\right]\left[\begin{array}{c} x_{1}\\ y_{1}\\ 1 \end{array}\right]\Leftrightarrow p_{2}=Hp_{1},$$

The matrix can then be expanded as:

(3) $$\{\begin{array}{c} x_{2}=H_{11}x_{1}+H_{12}y_{1}+H_{13}\\ y_{2}=H_{21}x_{1}+H_{22}y_{1}+H_{23}\\ 1=H_{31}x_{1}+H_{32}y_{1}+H_{33} \end{array},$$

where:

(4) $$\{\begin{array}{c} x_{2}\left(H_{31}x_{1}+H_{32}y_{1}+H_{33}\right)=H_{11}x_{1}+H_{12}y_{1}+H_{13}\\ y_{2}\left(H_{31}x_{1}+H_{32}y_{1}+H_{33}\right)=H_{21}x_{1}+H_{22}y_{1}+H_{23} \end{array},$$

Equation (4) can be further converted to:

(5) $$\{\begin{array}{c} x_{2}\left(H_{31}x_{1}+H_{32}y_{1}+H_{33}\right)-\left(H_{11}x_{1}+H_{12}y_{1}+H_{13}\right)=0\\ y_{2}\left(H_{31}x_{1}+H_{32}y_{1}+H_{33}\right)-\left(H_{21}x_{1}+H_{22}y_{1}+H_{23}\right)=0 \end{array},$$

Equation (5) is then rewritten as:

(6) $$a_{x}^{T}h=0,$$

(7) $$a_{y}^{T}h=0,$$

where

(8) $$h={\left(H_{11},H_{12},H_{13},H_{21},H_{22},H_{23},H_{31},H_{32},H_{33}\right)}^{T},$$

(9) $$a_{x}={\left(-x_{1},-y_{1},-1,0,0,0,x_{2}x_{1},x_{2}y_{1},x_{2}\right)}^{T},$$

(10) $$a_{y}={\left(0,0,0,-x_{1},-y_{1},-1,y_{2}x_{1},y_{2}y_{1},y_{2}\right)}^{T}$$

The last element of the homography matrix H is normalized to 1. Since the homography matrix H has eight unknown variables, at least four pairs of matching points (any three points are noncollinear) are needed to obtain the matrix for the two images. Usually, enough homonymous matching points can be obtained in adjacent spectral bands. Since the number of extracted keypoints is affected by the image features of ground objects, about 5000 to 100,000 matching points were obtained for each image in this study. The triangulation in the absolute registration process will be denser with more matching points, and higher registration accuracy will be produced. The least-squares method is used to estimate the homography matrix H in Equation (11).

(11) Ah=0,

where

(12) $$A=\left(\begin{array}{c} \begin{array}{c} a_{x_{1}}^{T}\\ a_{y_{1}}^{T} \end{array}\\ \vdots \\ \begin{array}{c} a_{x_{N}}^{T}\\ a_{y_{N}}^{T} \end{array} \end{array}\right),$$

2.  Matching point adjustment

**Figure 5:**
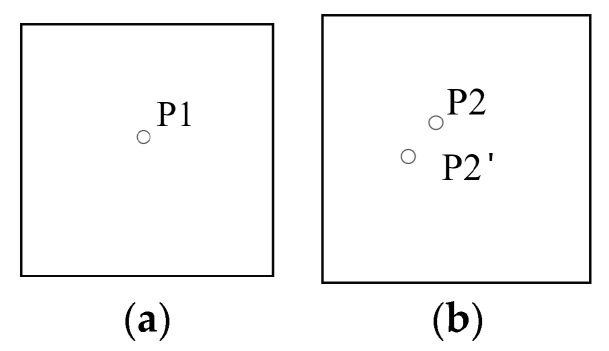
Schematic diagram of the adjustment for matching points. (**a**) Reference band. (**b**) Band to be registered.

The coordinates of each matching point for the reference band can be predicted in the sensed image using the estimated homography matrix H. The difference in the *x* and *y* directions and the root mean square (RMS) is calculated based on the predicted values and the actual matching point, respectively. Figure 4 displays a schematic diagram of adjustments for the matching points. In Figure 5, P1 and P2 are the matching point pairs and $P2_{}^{\prime }$ is the predicted position of P1 on the sensed image, calculated using the transformation matrix H. The root mean square error of the matching point pair is calculated using Equation (13), and the matching point pairs with a root mean square error greater than one are eliminated. This process is repeated until the total root-mean-square error of all matching point pairs (e.g., Equation (14)) is less than one pixel.

(13) $$R_{MSE}\left(i\right)=\sqrt{\Delta x^{2}+\Delta y^{2}},$$

(14) $$R_{MSE}=\sqrt{\frac{1}{N}\sum_{i=1}^{N}\left[R_{MSE}{\left(i\right)}^{2}\right]},$$

### 3.2. Absolute Registration

#### 3.2.1. Delaunay Triangulation Construction

The Delaunay triangulation for a set of points in a two-dimensional domain has two important properties. The first is an empty circumcircle property, which means that a circle circumscribing any Delaunay triangle does not contain any other input points in its interior. The second is a maximum-minimum angle property, which means that the Delaunay triangulation maximizes the minimum angle in the plane. The commonly used Delaunay triangulation construction algorithms include the incremental insertion method, triangulation growth method, and the divide and conquer method. In this study, the divide and conquer method was employed to construct the Delaunay triangulation.

**Figure 6:**
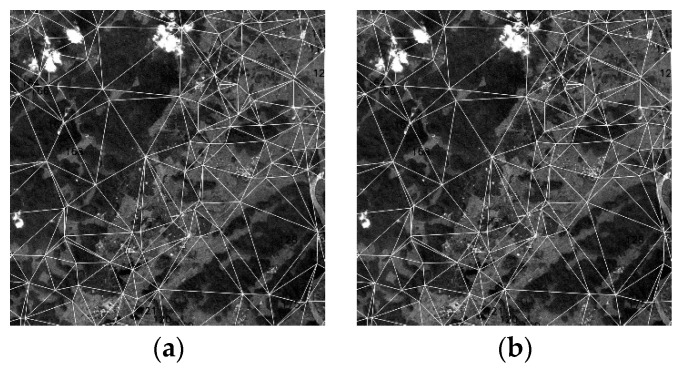
Delaunay triangulation. (**a**) Reference band (subset). (**b**) Band to be registered (subset).

Delaunay triangulation is created using the matching points obtained from relative registration (Figure 6). In this step, triangulation is constructed for the matching points in the reference band. An affine transformation relationship is derived based on each small triangle vertex in the reference band and the corresponding matching points in the sensed band. The equation is given as:

(15) $$\{\begin{array}{c} X=a_{1}x+b_{1}y+c_{1}\\ Y=a_{2}x+b_{2}y+c_{2} \end{array},$$

where $a_{1}$, $b_{1}$, $c_{1}$, $a_{2}$, $b_{2}$, and $c_{2}$ represent the affine transformation coefficients for the two triangles; (*x*, *y*) are the coordinates for the matching point in the reference band; and (*X*, *Y*) are the coordinates for the matching points in the image to be registered.

#### 3.2.2. Affine Transformation Relationship Transfer Strategy

The affine transformation relationship for adjacent bands can be transferred to perform registration for all other bands after a spectral band is selected as the reference band. The criteria for reference band selection is that the band should represent the texture characteristics of ground objects and have high contrast. The reference band can be chosen by analyzing the signal-to-noise ratio and range of spectral bands. Generally, the middle or red band is selected as the reference band. There are two situations in the registration process, including the registration of bands that are adjacent and non-adjacent to the reference band. In the first case, the differential correction method based on dense Delaunay triangulation is directly used for registration. In the second case, the registration of non-adjacent bands is completed by combining the transferred affine transformation relationship of adjacent bands with a differential correction from dense Delaunay triangulation.

**Figure 7:**
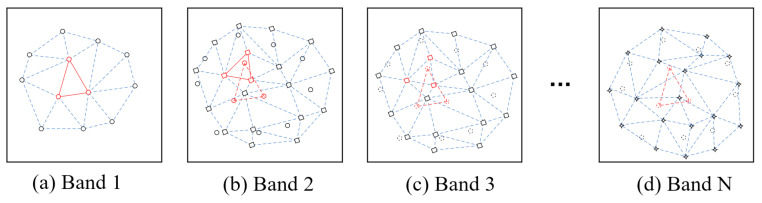
Full-spectrum registration diagram.

Figure 7 displays a schematic diagram of full-spectrum registration. In the figure, the reference image is Band 1. The solid circle points in Figure 7a,b are the matching point pairs in Band 1 and Band 2. The diamond-shaped points in Figure 7b,c are the matching point pairs in Band 2 and Band 3. The quadrangular points in Figure 7d are the matching point pairs in Band N-1 and Band N. The dotted circle points in Figure 7c,d are the matching points (solid circle points) in (b) in Band 3 and Band N.

The registration steps for Band 1 and Band 2 are described below.

Step 1: The matching points in Band 1 (the solid circled points in Figure 7a) are used to construct a dense Delaunay triangulation D1.

Step 2: The affine transformation coefficients are calculated using Equation (15). This is based on the three vertices of the small triangle in D1 (the red solid triangle vertices), and the corresponding matching points in Band 2 (the red dashed triangle vertices in Figure 7b).

Step 3: An affine transformation is performed on the internal points of the small triangles in D1. The correct coordinates are calculated for the internal points in Band 2. Nearest neighbor interpolation, bilinear, or bicubic interpolation is then used to calculate new pixel values.

Step 4: Steps (2) and (3) are repeated until all triangle calculations are complete.

The registration of Band 3 and Band 1 can be used as an example. The following steps describe registration in the second case.

Step 1: The matching points for Band 2 and Band 3 (the diamond point in Figure 7b) are used to construct a dense Delaunay triangulation D2.

Step 2: Equation (15) is used to calculate the affine transformation coefficients. This calculation is based on the small triangle vertices in D2 (the red diamond points), and the corresponding matching points in Band 3 (the red diamond points in Figure 7c).

Step 3: The coordinates for the matching points between Band 1 and Band 2 (the red solid circle point in Figure 7b) are calculated for Band 3 (the red dashed circle point in Figure 7c) based on the affine transformation relationship.

Step 4: Steps (2) and (3) are repeated until all the matching points in Band 1 and Band 2 (the solid circle point in Figure 7b) are calculated for Band 3 (the dotted circle point in Figure 7c).

Based on the above steps, the coordinates of the matching points for Band 1 and Band 2 are derived in Band 3 using the transfer strategy based on the affine transformation relationship between adjacent bands. Thus, Band 3 can be regarded as being adjacent to Band 1. The registration of Band 3 can then be performed using the differential correction method based on dense Delaunay triangulation. The coordinates of matching points in Band 1 and Band 2 can be calculated for Band N, which allows for the registration of Band N.

**Figure 8:**
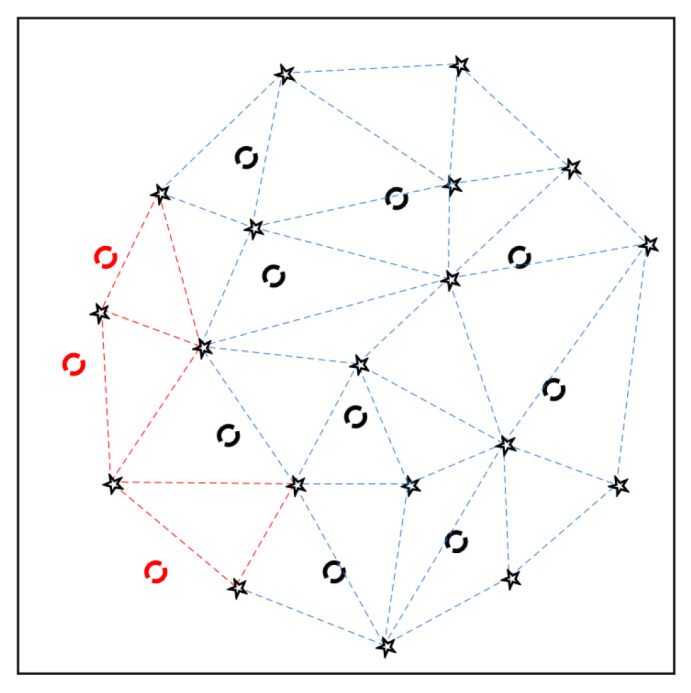
Schematic diagram of matching points outside Delaunay triangulation.

The constructed Delaunay triangulation will differ due to the different positions and number of feature points that are extracted for each band. The position of the calculated matching points in Band 1 can be outside the Delaunay triangulation in Band M (e.g., the red dotted circles in Figure 8). In this case, the center of gravity of each triangle was calculated to find the triangle closest to the matching point. This is represented as the red dotted triangle in Figure 8. The affine transformation relationship that is established by the closest triangle is used for resampling pixels. To reduce registration errors which might be caused by mixed pixels, a bilinear resampling operation was performed when applying the affine transformation.

### 3.3. Evaluation Criteria

#### 3.3.1. Coordinate Deviation of Homonymous Points

The coordinate deviation for homonymous matching points was calculated to estimate the registration accuracy between adjacent bands using the following Equation:

(16) $$\{\begin{array}{c} m_{X}=\sqrt{\frac{\sum_{i=1}^{N}{\left(X_{b}-X_{w}\right)}_{i}^{2}}{N}}\\ m_{Y}=\sqrt{\frac{\sum_{i=1}^{N}{\left(Y_{b}-Y_{w}\right)}_{i}^{2}}{N}}\\ m_{A}=\sqrt{m_{X}^{2}+m_{Y}^{2}} \end{array},$$

where ($X_{b},$ $Y_{b}$) are the coordinates of a matching point in the reference band; ($X_{w}$, $Y_{w}$) are the coordinates of a matching point in the band to be registered; $m_{X}$, $m_{Y}$, and $m_{A}$ are the accuracy values in the X and Y direction and the total accuracy of the registered image, respectively; and N is the number of matching points used in the accuracy assessment.

Overall, 20 evenly distributed homonymous points were manually selected for the accuracy assessment of non-adjacent bands. Equation (16) was used to evaluate the registration accuracy for non-adjacent bands.

#### 3.3.2. Similarity Measures

A block strategy was used to generate the similarity measures for two bands. First, the two bands were divided into blocks, whose sizes were determined based on the size of the image. Generally, the block size can be set as 50 × 50, 100 × 100, or 200 × 200 pixels, and it was set as 100 × 100 pixels in this study. The similarity measures for corresponding blocks were then calculated to evaluate the distribution of accuracy in the entire image. In this study, two commonly used similarity measures, structural similarity index (SSIM) and cosine similarity (CS), were selected for the accuracy assessment of image registration.

The structural similarity measure is an objective image quality evaluation standard that is in line with the characteristics of the human visual system [34]. This indicator measures image similarity based on three aspects: brightness (mean), contrast (variance), and structure. Equation (17) is given as:

(17) $$SSIM\left(P,Q\right)=l{\left(P,Q\right)}^{\alpha }\cdot c{\left(P,Q\right)}^{\beta }\cdot s{\left(P,Q\right)}^{\gamma },$$

where

(18) $$l\left(P,Q\right)=\frac{2u_{P}u_{Q}+c_{1}}{u_{P}^{2}+u_{Q}^{2}+c_{1}},$$

(19) $$c\left(P,Q\right)=\frac{2\sigma_{P}\sigma_{Q}+c_{2}}{\sigma_{P}^{2}+\sigma_{Q}^{2}+c_{2}},$$

(20) $$s\left(P,Q\right)=\frac{2\sigma_{PQ}+c_{3}}{\sigma_{P}\sigma_{Q}+c_{3}},$$

(21) $$\sigma_{PQ}=\frac{1}{N-1}\sum_{i=1}^{N}\left(P_{i}-\mu_{P}\right)\left(Q_{i}-\mu_{Q}\right),$$

where *P* and *Q* are the reference image and the image to be registered, respectively; $\mu_{P}$, $\mu_{Q}$, $\sigma_{P}^{2}$, $\sigma_{Q}^{2}$, and $\sigma_{PQ}$ represent the mean, variance, and covariance of images *P* and *Q*, respectively; $c_{1}$, $c_{2}$, and, $c_{3}$ are small normal numbers that are used to avoid instability due to the zero denominator in Equations (18)–(20); the parameters α, β, and γ are all greater than zero and are used to adjust the proportions of the three components in the model; and the value range of SSIM is [0,1], with larger values indicating higher image similarities. The following equation is given when α = β = γ = 1 and $c_{3}$ = $c_{2}/2$:

(22) $$SSIM\left(x,y\right)=\frac{\left(2u_{x}u_{y}+c_{1}\right)\left(2\sigma_{xy}+c_{2}\right)}{\left(u_{x}^{2}+u_{y}^{2}+c_{1}\right)\left(\sigma_{x}^{2}+\sigma_{y}^{2}+c_{2}\right)},$$

Cosine similarity measures the similarity between two vectors by measuring the cosine value of their inner product space, which is suitable for the vector comparison of any dimensions [35]. Digital images contain more feature codes that belong to high-dimensional space, and thus represent the application domain of the cosine similarity algorithm. The satellite image is regarded as a vector, and the cosine distance between the vectors is calculated to characterize the similarity between two images (Equation (23)) as:

(23) $$\cos\left(\theta \right)=\frac{\sum_{i=1}^{n}\left(X_{i}\times Y_{i}\right)}{\sqrt{\sum_{i=1}^{n}{\left(X_{i}\right)}^{2}}\times \sqrt{\sum_{i=1}^{n}{\left(Y_{i}\right)}^{2}}},$$

where $X_{i}$ and $Y_{i}$ represent the image vectors of the reference and sensed image, respectively. The normalized cosine similarity values range from 0 to 1, with larger values representing higher image similarity.

## 4. Results and Discussion

### 4.1. Experiment Results

**Figure 9:**
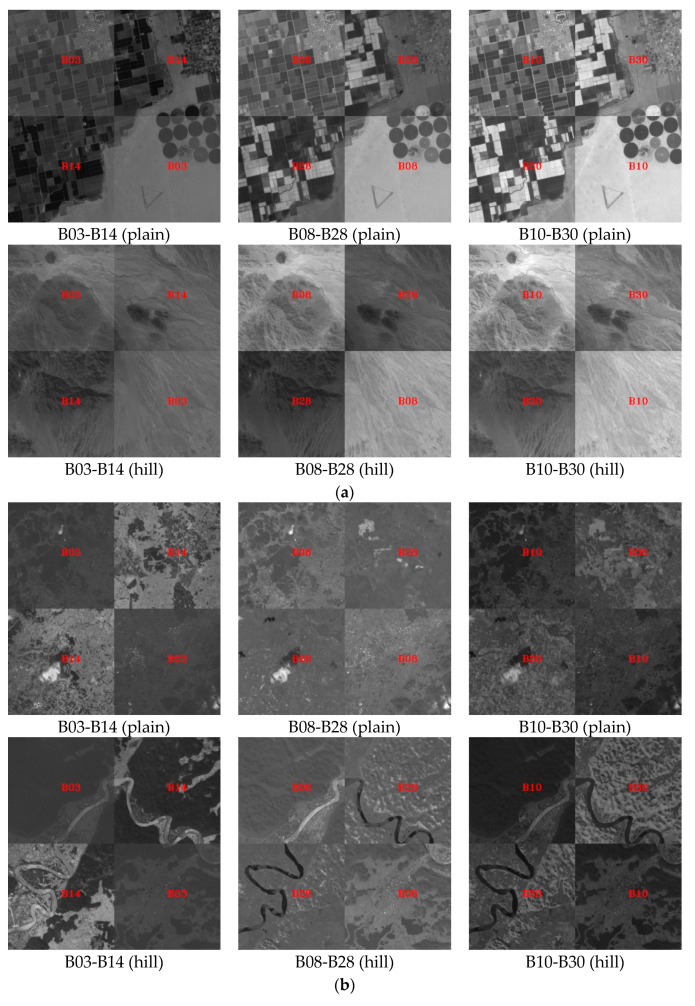

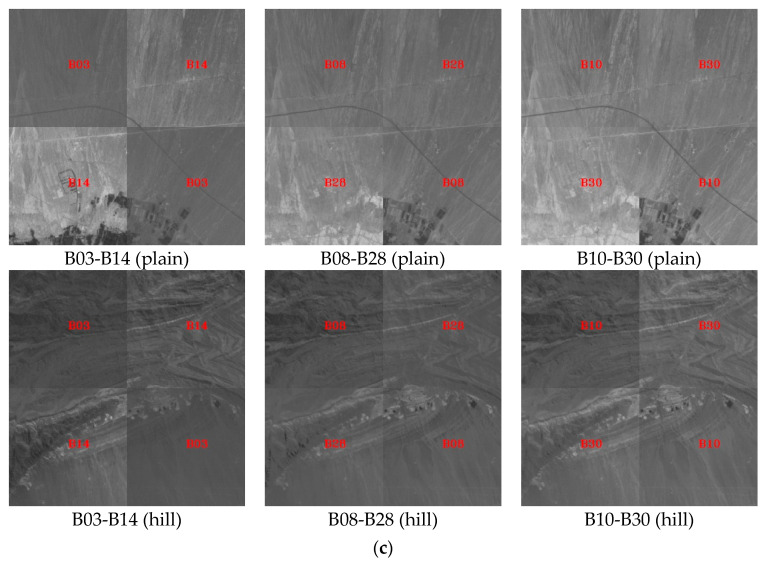
Local comparison of registration results between different bands. (**a**) OHS–Arizona, USA; (**b**) OHS–Guangxi, China; (**c**) OHS–Xinjiang, China.

**Figure 10:**
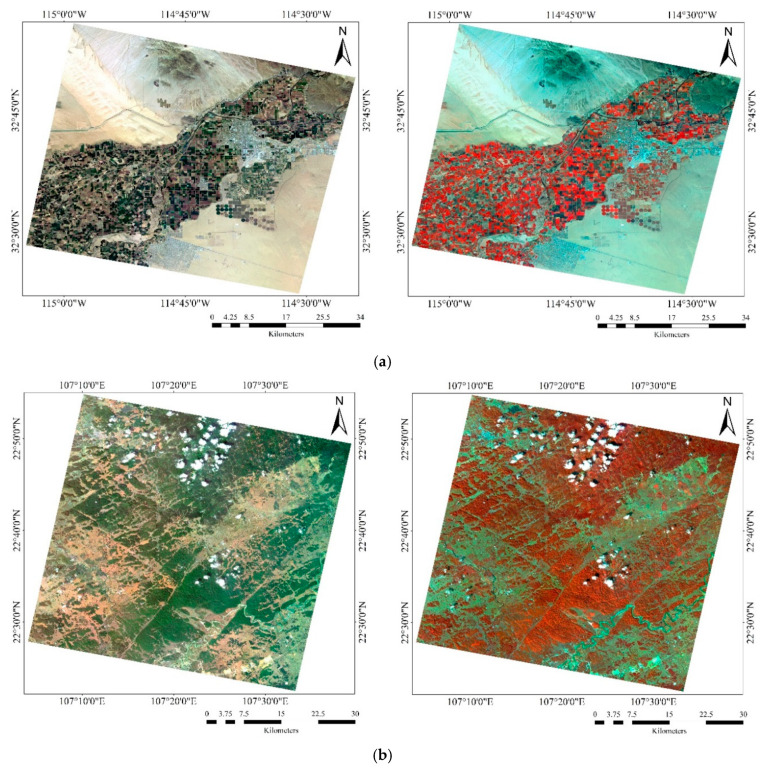

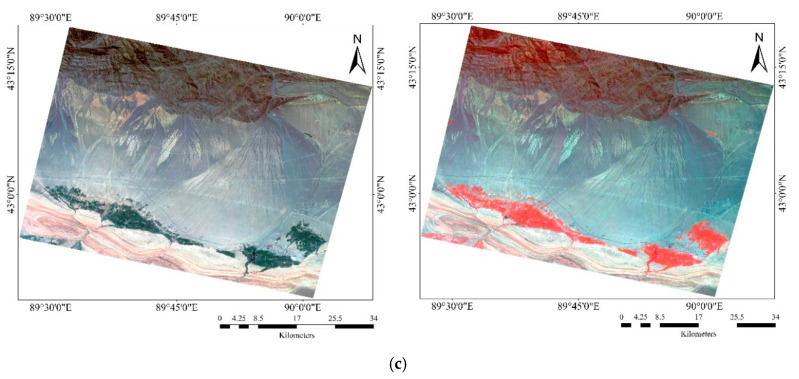
True and false-color composite images after registration. Left column: true color (R: B14, G: B08, B: B03), Right column: false-color (R: B28, G: B08, B: B03). (**a**) OHS–Arizona, USA; (**b**) OHS–Guangxi, China; (**c**) OHS–Xinjiang, China.

In the experiment, B15 (686 nm) was selected as the reference image to perform registration for all other bands. Several of the most common bands were selected for registration accuracy analysis due to the large number of spectral bands. These bands included: B03 (490 nm) and B14 (670 nm), B08 (560 nm) and B28 (880 nm), and B10 (596 nm) and B30 (910 nm). The two bands were overlaid in the form of a checkerboard to verify the registration algorithm. Figure 9 displays the registration results using the proposed method. Visually, misalignment was not identified for rivers, roads, fields, and other land cover features between bands. The overall registration result was satisfactory. Figure 10 further displays the true color (R: B14, G: B08, B: B03) and false-color (R: B28, G: B08, B: B03) composite images after registration.

### 4.2. Comparison with IOEM Method

**Figure 11:**
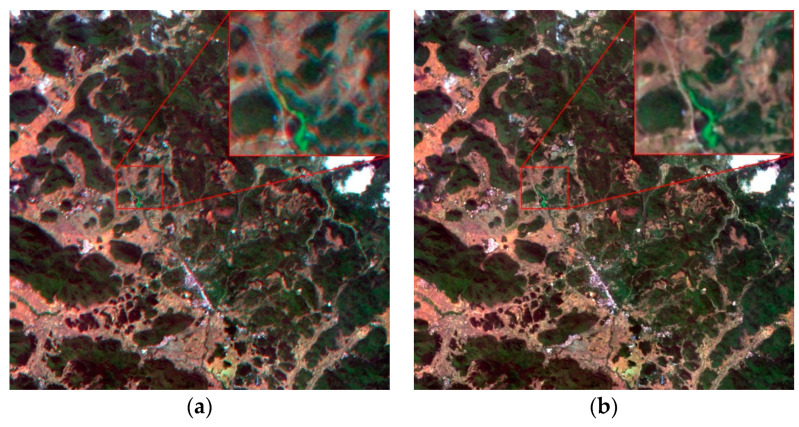

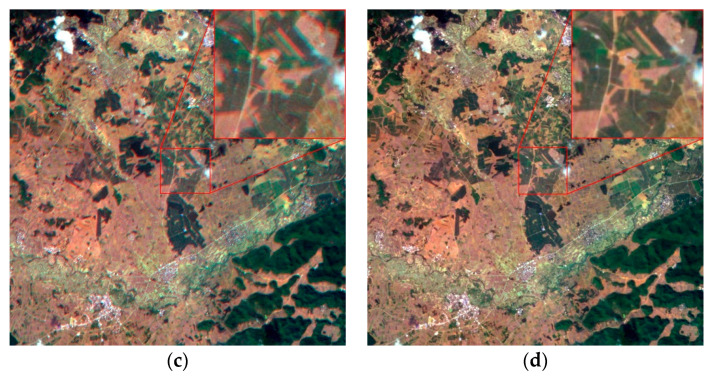
Comparison of registration results using IOEM and the proposed method. (**a**) IOEM method; (**b**) Proposed method; (**c**) IOEM method; (**d**) Proposed method.

The proposed method was compared with the IOEM method [31]. The IOEM method uses the external and internal parameter error from the hyperspectral camera for on-orbit calibration. Moreover, the IOEM method uses the established internal orientation element model to complete the registration of all spectral bands. Figure 11 illustrates the registration results for the IOEM method and the proposed method for the dataset of OHS-Guangxi, China. The true color composite images produced by the IOEM method exhibited an obvious ghost-shadow phenomenon. Conversely, the true color composite imagery produced by the proposed method clearly showed ground objects with sharper edges and details, and thus featured a better registration accuracy.

**Figure 12:**
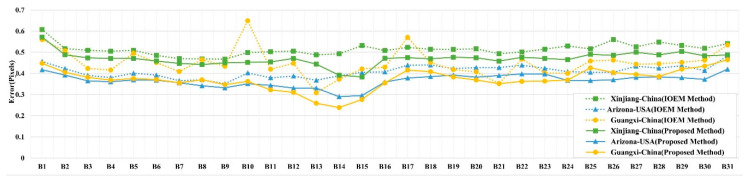
Registration accuracy of adjacent bands using different methods.

The homonymous points were extracted from the adjacent spectral bands after registration. The coordinate displacement between homonymous points was then calculated using Equation (16) to estimate the registration accuracy between adjacent spectral bands. Figure 12 shows the registration accuracy of adjacent bands when using the two methods. The maximum error produced by the IOEM method was 0.65 pixels and the minimum error was 0.30 pixels. However, the maximum error produced by the proposed method was 0.57 pixels and the minimum error was 0.23 pixels. Different accuracies were achieved for the three experimental images. Relatively high errors were revealed in the image covering Xinjiang, China. Since there are large deserts and fewer texture details in the image of Xinjiang, fewer feature points were detected than the other two images. A relatively small number of correctly matched keypoints may lead to a decrease in registration accuracy.

In total, 20 uniformly distributed homonymous points were manually selected for registration accuracy assessment between non-adjacent bands in B03, B14, B08, B28, B10, and B30, respectively. The coordinate deviation between homonymous points was calculated using Equation (16). The registration accuracy between non-adjacent bands was greater than one pixel for the IOEM method. The registration accuracy between non-adjacent bands was located at the sub-pixel level in the proposed method, as shown in Table 3. Lower RMSE values of the proposed method indicate that it can obtain higher registration accuracies than IOEM method.

**Figure 13:**
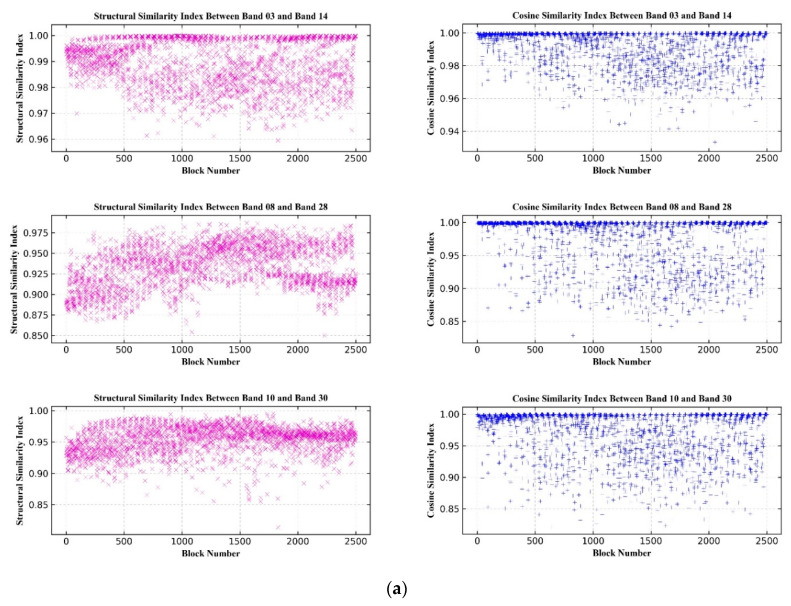

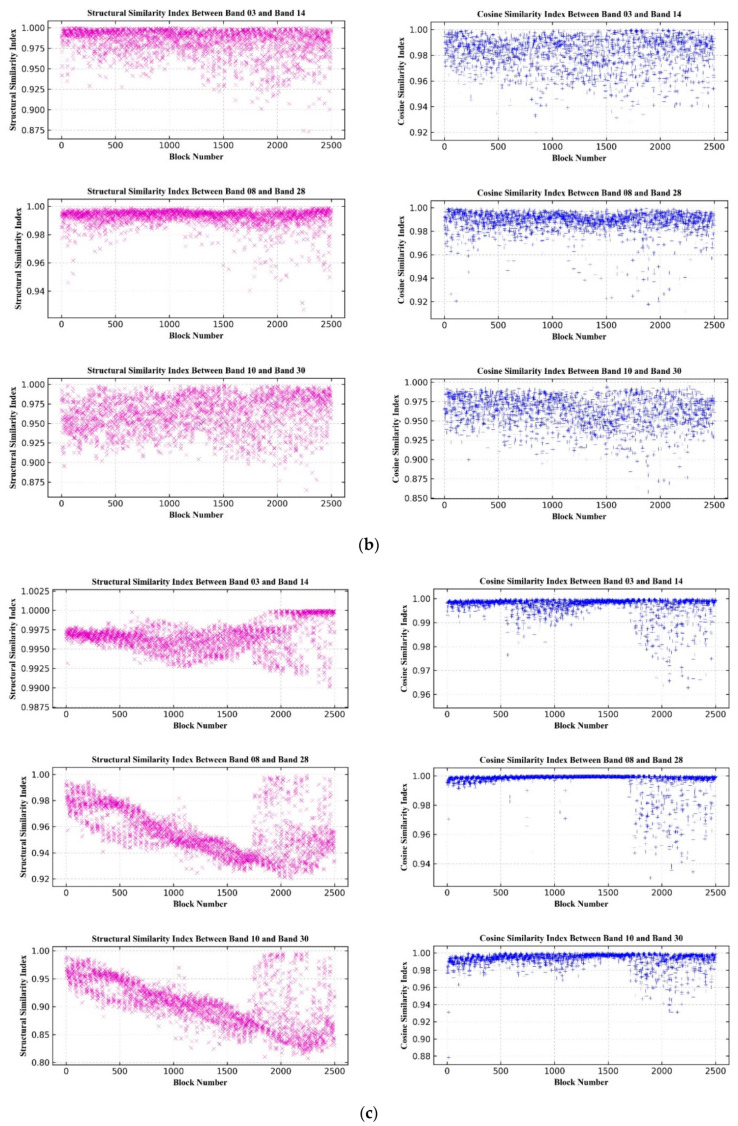
Similarity measures for the registered spectral bands. (**a**) OHS–Arizona, USA; (**b**) OHS–Guangxi, China; (**c**) OHS–Xinjiang, China.

In this study, the structural similarity measure and cosine similarity measure were used to evaluate the similarity between registered spectral bands. The similarity measures were calculated with a size of 100 × 100 pixels. As shown in Figure 13, the structural and cosine similarity measures between B03 and B14, B08 and B28, and B10 and B30 were all greater than 0.80 for the three experimental datasets. The distribution of structural similarity index values is more concentrated in the Guangxi image than the other two experimental sites, while the cosine similarity index values are more concentrated in the Xinjiang image (Figure 13). This might be because the two indices measure the similarity of images in different aspects. Therefore, similarity measures should be used conjunctively to gain a comprehensive understanding of the registration results.

The registration results can help facilitate the application of Zhuhai-1 hyperspectral imagery to various fields. Hyperspectral imagery can capture the reflectance at hundreds of wavelengths and enable the analysis of the spectra of materials on the Earth’s surface. Registered Zhuhai-1 hyperspectral imagery can be used for urban land cover mapping, mineral mapping, and forest and plant species identification [36]. The full-spectrum registration approach can also be compared with other approaches (e.g., deep-learning based methods) for further improvement [37,38]. With the capability of capturing increasingly complex image characteristics, deep convolutional neural networks have been applied to feature extraction for image registration and outperformed traditional feature detectors with fewer outliers [39,40]. The proposed method can be adjusted and applied to hyperspectral images acquired by other satellite or airborne hyperspectral sensors. It also has great potentials for registration of satellite images captured at different times, from different viewpoints or from different sensors, to facilitate applications such as change detection and urban sprawl monitoring [41].

## 5. Conclusions

In this paper, a method was developed to address the issue relating to the accurate registration of spectral bands in Zhuhai-1 satellite hyperspectral imagery. A full-spectrum registration method was proposed that combines the transference strategy for the affine transformation relationship between adjacent bands with the differential correction from dense Delaunay triangulation. This method uses the affine transformation relationship between adjacent bands to establish the same relationship between non-adjacent bands. This method was used to conduct registration of non-adjacent bands with large gray variations. Moreover, dense Delaunay triangulation was constructed to establish multiple affine transformation relations for triangular differential correction and to eliminate the influence of local distortion on registration accuracy caused by terrain undulation. Finally, an experiment was conducted using three Zhuhai-1 satellite hyperspectral images covering different topographies. The experiment showed that the proposed method could obtain accurate and robust registration results. The registration accuracy between adjacent spectral bands ranged from 0.2 to 0.6 pixels. The registration accuracy for non-adjacent bands reached a sub-pixel level, and the resulting structural and cosine similarity measures were both greater than 0.80. 

## Figures and Tables

**Table 1:** Orbita hyperspectral satellite (OHS) and payload parameters.

	Parameters	Index
Satellite	Total satellite mass	67 kg
	Orbit height	500 km
	Orbit inclination angle	98°
	Regression cycle	2.5 days
Payload	Detector size	4.25 µm
	Field of view (FOV)	20.5°
	Spectral range	400–1000 nm
	Quantitative level	12 bits
	Band number	32
	Signal Noise Ratio (SNR)	≥300 dB
	Ground sample distance	10 m
	Spectral resolution	2.5 nm
	Ground swath	150 km

**Table 2:** Image registration experiment data.

Name	Long/Lat	Imaging Time	Minimum/Maximum Elevation(m)	Lateral Angle
OHS-Arizona-USA	−114.7/32.6	2020-04-02	18/633	−12.688
OHS-Guang Xi-China	107.4/22.6	2020-04-15	93/1023	−1.88
OHS-Xin Jiang-China	89.8/43.0	2020-05-29	−60/3801	−5.703

**Table 3:** Registration accuracy between non-adjacent bands.

Name	Method	RMSE (Pixel)			Mean RMSE (Pixel)
		B03-B14	B08-B28	B10-B30	
OHS-Arizona-USA	IOEM	1.68	2.02	1.60	1.77
	Our method	0.68	0.39	0.42	0.50
OHS-Guangxi-China	IOEM	2.28	3.34	3.76	3.13
	Our method	0.52	0.54	0.53	0.53
OHS-Xinjiang-China	IOEM	1.00	1.03	1.24	1.09
	Our method	0.58	0.72	0.49	0.60

## Software Availability

The codes of the proposed registration method are available from the corresponding author upon reasonable request.
